# Azithromycin mass drug administration to reduce childhood mortality in humanitarian crises

**DOI:** 10.1371/journal.pgph.0006684

**Published:** 2026-07-10

**Authors:** Gregory Barnsley, Matthew Coldiron, Neal Russell, Aula Abbara, Douglas James Noble, Stefan Flasche, Kevin van Zandvoort, Bhargavi Rao

**Affiliations:** 1 Department of Infectious Disease Epidemiology, London School of Hygiene and Tropical Medicine, London, United Kingdom; 2 City St George's, University of London, London, United Kingdom; 3 Médecins Sans Frontières, London, United Kingdom; 4 UNICEF HQ, New York, New York, United States of America; 5 Charité Center for Global Health, Charité-Universitätsmedizin Berlin, Berlin, Germany; 6 Department of Global Health and Development, London School of Hygiene and Tropical Medicine, London, United Kingdom; Universiti Kebangsaan Malaysia, MALAYSIA

## Abstract

Azithromycin mass drug administration (MDA) can reduce under-5 mortality in stable but high-mortality settings, yet its potential in humanitarian crises remains undefined. This review proposes that azithromycin MDA could support existing humanitarian interventions and help address mortality from respiratory and gastrointestinal infections in displaced populations. Mortality rates in crises frequently exceed WHO recommended thresholds for initiating MDA. Operationally, the MDA is feasible, low-cost, and synergises with existing relief efforts like vaccination. While antimicrobial resistance (AMR) is a serious concern, the proposed use, likely a single round or short-term intervention during acute emergencies, may mitigate long-term resistance selection compared to routine biannual dosing. The potential to rapidly reduce excess mortality may outweigh the AMR risks. Azithromycin MDA represents a promising new tool for humanitarian health, though evidence-based protocols and resistance monitoring strategies must be developed through further research.

## Introduction

An estimated 122.6 million people were displaced due to crises caused by conflict, climate, persecution, or natural disasters by the end of 2023, 40% of them children [1]. Crises are associated with substantial excess mortality, often more than doubling the pre-crisis mortality rates [2]. Whilst disasters and conflicts themselves may directly cause an increase in mortality through injuries, displaced populations are also at a higher risk of communicable disease [3], generally due to the lack of water and sanitation, overcrowding, increased mixing with disparate population groups, co-morbidities [4], and disruptions to control programmes like vaccinations [5,6]. This burden is typically highest amongst young children, who are most vulnerable to infectious diseases and severe acute malnutrition, which is associated with increased disease severity [7].

Humanitarian health response aims to reduce the excess crisis-attributable mortality and morbidity. During the acute phase of a crisis, where mortality is frequently greatest, the typical priority is improving sanitation and treating acute malnutrition [8]. Some vaccine-preventable diseases, such as measles, are also addressed via reactive or prospective mass vaccination campaigns [9].

Mass drug administration (MDA) is the single or semiregular symptom-agnostic administration of a drug to a large (sub-) population to treat existing infections and prevent new infections. Azithromycin is a macrolide antibiotic, often prescribed for bacterial upper and lower respiratory infections such as pneumonia and otitis media, and some sexually transmitted infections [10]. Azithromycin MDA was introduced for trachoma control in children and adults [11]. After trials demonstrated significant reductions in childhood mortality, the WHO gave a conditional recommendation for its use in reducing all-cause childhood mortality in sub-Saharan African settings where infant mortality is greater than 60/1000 live births or under-5 mortality (u5MR) is greater than 80/1000 live births [12].

In this narrative review, we analyse evidence to explore the potential for the use of azithromycin MDA in acute humanitarian crises, discuss the risks and challenges, and establish a future research agenda for this potential intervention.

## Search strategy

As no randomised trial, observational, or implementation study directly on the use of azithromycin MDA to improve mortality in a humanitarian setting was identified by a preliminary search (see S1 Appendix), a systematic or scoping review on this question would not have been feasible. We opted to perform a narrative review to better capture the heterogeneities of the evidence around azithromycin MDA. We aimed to synthesize research from four overarching domains: evidence on the impact of azithromycin MDA on mortality in any setting, evidence on the mechanism behind the observed reduction in mortality (i.e., which diseases and pathogens are affected), the operational feasibility of other MDA programmes in crisis settings, and the risk of antimicrobial resistance from azithromycin MDA.

The initial literature search was conducted using PubMed and Google Scholar up to the 3rd of November 2025. We searched using the terms “azithromycin mass drug administration”, “azithromycin childhood mortality”, “mass drug administration crisis emergency”, “mass drug administration displaced population”, and “azithromycin mass drug administration macrolide resistance”. To get a more comprehensive picture of trial data, we also searched ClinicalTrials.gov for trials involving azithromycin (see S1 Appendix) and reviewed the publications lists to identify relevant sub-studies.

Articles were included if they were published in English and examined the impact of population-level azithromycin MDA (or similar interventions, such as treatment during routine visits) on childhood mortality or more specific diseases and pathogens, if they explored the impact of azithromycin MDA on antibiotic resistance, or if they evaluated the feasibility of MDA in displaced populations or settings where routine health services had been disrupted. Existing systematic reviews on these topics were preferred. Grey literature was included where relevant to the discussion, such as treatment guidelines for azithromycin MDA [13] and WHO recommendations [11].

## Rationale for the use of azithromycin MDA in emergencies

A reduction in all-cause childhood mortality was observed in cohorts undergoing azithromycin MDA to control trachoma [14]. This observation led to the MORDOR study [15], a cluster randomised controlled trial, which demonstrated that a round of azithromycin MDA every 6 months (semi-annual) in children under five reduced childhood mortality by 13.5% (6.7% - 19.8% 95% CI). The WHO has since given a conditional recommendation for the biannual MDA of azithromycin to children from 1 to 11 months to reduce childhood mortality in sub-Saharan African countries with high rates of infant or under-5 childhood mortality [12]. Subsequent trials have compared targeting infants to children in Niger [16], quarterly to biannual intervals in Mali [17], including azithromycin in SMC in Burkina Faso and Mali [18], and expanding existing trachoma MDA programmes in Côte d'Ivoire [19]. Currently, active and/or recruiting trials are investigating: i) the inclusion of azithromycin MDA in child health days in Burkina Faso [20]; ii) the inclusion of MDA in Essential Programme on Immunization (EPI) activities in Sierra Leone [21]; iii) the impact of MDA on mortality; morbidity, and resistance in Nigeria [22]; and iv) the national scale-up of MDA and stopping criteria in Niger [23]. Burkina Faso and Mali [24] currently plan to introduce or are introducing this intervention outside of clinical trials.

Displaced populations often experience substantial increases in childhood mortality [25]. The WHO conditional recommendation is for azithromycin MDA in contexts where u5MR exceeds 80 per 1000 live births. Generally, mortality in humanitarian settings is reported per person-time to better capture short-term changes in the mortality rate. We can approximately convert the WHO threshold to 0.46 deaths per 10,000 person days (see S1 Appendix) [12]. This approximate threshold was exceeded by the estimated u5MR in all 25 complex emergencies identified by a review covering 1991–2002 [26]. A modelling study also collected u5MR estimates from humanitarian settings from 1999 to 2020 [27]. Although they did not report individual estimates, from their pooled summaries we can estimate that this threshold would have been exceeded 69%, 75%, and 72% of the time in non-displaced, IDP, and refugee populations experiencing a crisis. Furthermore, in the MORDOR trial, the most substantial (and only significant), impact on mortality was observed in Niger, the setting among the three trial sites with the highest u5MR at 0.75 per 10,000 person days (exceeded in 62%, 72%, and 58% of the historic crises) [15]. It seems likely, therefore, that azithromycin MDA may have a greater impact in settings with higher mortality, such as humanitarian crises.

Direct translation of the benefits and risks of azithromycin MDA from non-crisis to crisis settings may not be entirely appropriate; nonetheless, it is unlikely that research to guide this policy will be carried out in emergencies in the coming years. Policymakers are left to use their best judgment and assess based on similar interventions [28]. For example, MDA-based interventions have been used in acute emergencies for malaria control. A review identified [29] two studies [30,31] of antimalarial MDA that found some evidence of decreases in malaria related endpoints but were limited by a lack of control arms, but also included modelling based evidence [32]. Despite the lack of clear evidence, antimalarial MDA for short-term burden reductions in emergencies or periods of disruption of healthcare services remains a conditional recommendation in the WHO malaria guideline [33]. Subsequent studies [34,35] have continued to show decreases in malaria related endpoints, but again have been limited by a lack of randomisation or control arms.

As azithromycin MDA has substantially reduced u5MR in settings with high childhood mortality, and other MDA programmes have been effectively deployed in humanitarian settings, azithromycin MDA for improving child survival could be considered for use in these crises. This short-term intervention may be particularly useful in the acute phase of a crisis and would complement longer-term interventions, such as improvements in nutrition; water, sanitation, and hygiene (WASH); or vaccinations.

## How does azithromycin MDA reduce child mortality?

A critical objection to using azithromycin MDA to reduce childhood mortality is the lack of clarity around the mechanism behind its impact on all-cause mortality [36]. A comparison of causes of death in the MORDOR site in Malawi [37] using verbal autopsies indicated potential reductions in pneumonia, diarrhoea, and HIV/AIDS-related mortality in the treated populations, though this study lacked power. However, a verbal autopsy analysis from the Niger site, with better statistical power, showed a reduction in deaths due to malaria, dysentery, meningitis, and pneumonia, and no statistically significant change for AIDS and diarrhoea related mortality, though both of these studies are limited by the inherent difficulties in verbally attributing cause of death. The Niger sites saw the largest decline in their under-5 mortality rate (18.1% (10.0% - 25.5%)) compared to the Malawi (5.7% (-9.7% - 18.9%)) and Tanzania (3.4% (-21.2% - 23.0%)) sites. This could be explained by Niger's greater baseline u5MR (27.5 against 9.6 and 5.5 per 1000 person-years) and higher proportion of under-5 deaths due to lower respiratory tract infections and diarrhoeal diseases (28.0% against 18.7% and 21.9%) [38]. Baseline levels of resistance to macrolides may also modulate impact, low levels of nasopharyngeal macrolide resistance were observed in the placebo arm of the Niger sites, 2.9% (0% - 6.1%), significantly lower than the baseline levels in the Tanzania and Malawi sites (see Fig 3).

**Fig 1:**
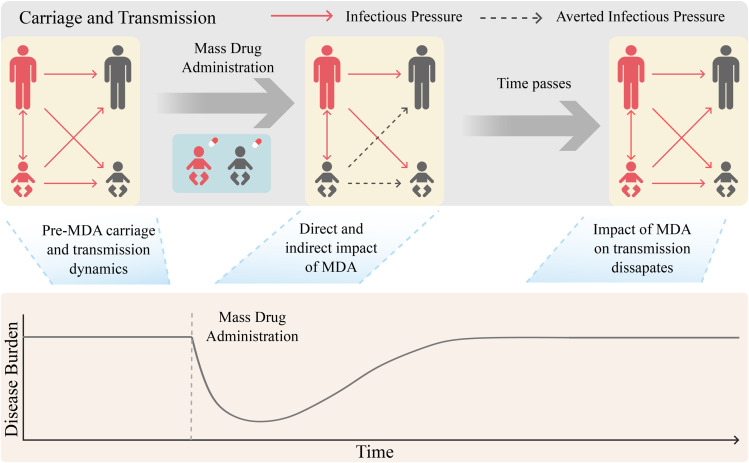
Demonstration of the impact of a round of azithromycin mass drug administration on disease carriage and transmission, and how this relates to disease dynamics. Arrows indicate infectious pressure, i.e., people an infected person might reasonably infect in the short term. Dashed arrows indicate averted infectious pressure; this transmission potential is lost due to the clearance of bacteria. From the baseline dynamics, MDA treats the child population, which temporarily clears carriage of bacteria. This also creates indirect effects on transmission, as previously infected children no longer exert an infectious pressure on adults or children. This reduction in carriage (and hence disease) wanes over time as the bacteria reestablish themselves in the treated population, and a similar dynamic to before MDA returns.

**Fig 2:**
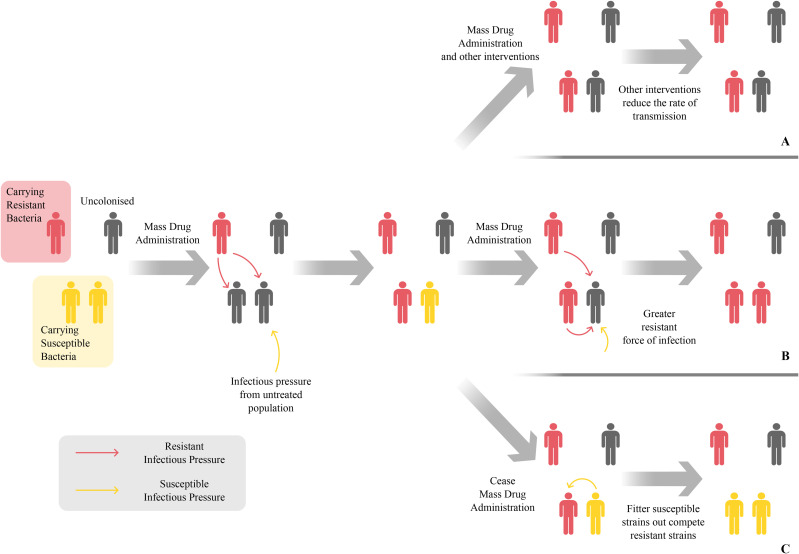
Conceptual diagram of the impact of mass drug administration of azithromycin on the carriage of antibiotic-resistant bacteria, assuming that macrolide resistance has a fitness cost. The initial round of MDA eliminates susceptible bacteria. Carriage dynamics return to the initial state with the importation of susceptible bacteria and resistant bacteria colonising the uncolonised hosts. A: If combined with other interventions that reduce transmission, then although the proportion of resistance is high, the prevalence of carriage of resistant bacteria is limited. B: If MDA is applied again, the susceptible bacteria are again cleared and resistant bacteria are able to colonise more hosts, meaning future rounds of MDA have less impact on carriage and there are higher rates of resistance. C: If MDA is ceased, susceptible bacteria outcompete resistant bacteria and eventually carriage returns to the pre-MDA dynamics of resistance.

**Fig 3:**
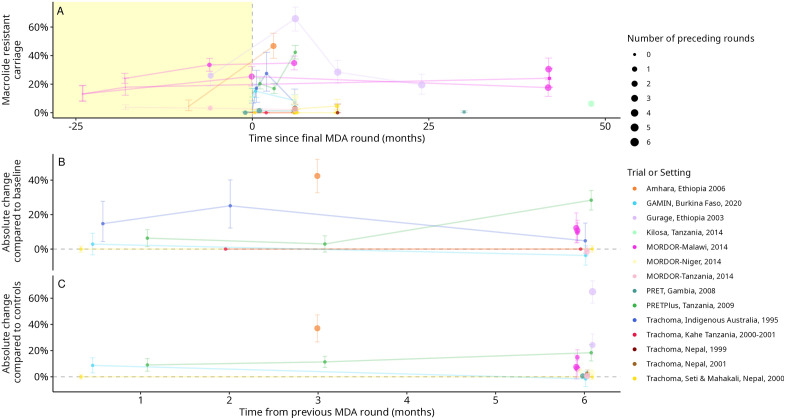
Observed impact of azithromycin mass drug administration on macrolide resistance in *Streptococcus pneumoniae.* **a)** Carriage of macrolide resistant pneumococcus in studies relative to the final mass drug administration of azithromycin round. **b)** Macrolide resistance in pneumococcus up to 6 months post mass drug administration of azithromycin, by change in percentage points relative to the baseline (always before the first MDA round) or relative to control groups given in each study.Colour indicates the trial or setting the points are derived from, point size indicates the number of previous MDA rounds. Lines connect measurements from the same cohorts in each study. Not all trials reported confidence intervals, for the sake of comparison all confidence intervals are Bayesian credible intervals (see S1 Appendix) based upon the number of samples for carriage and the number of tests for resistance reported in each study. Credible intervals should not be used to draw strong statistical conclusions, they simply are demonstrative of the sample sizes contributed by each study. Where studies report intermediate resistance, we do not include it as resistance. In studies where macrolide resistance was not reported, azithromycin or erythromycin resistance was used instead. Measurement points where pneumococcal carriage was not reported were excluded. Due to overlapping points, a jitter of ± 0.1 months was added to the results from each cohort [84].

Other trials support the notion that azithromycin MDA impacts infectious diseases. A cluster randomised trial in Niger compared the WHO recommendation of treating 1–11 month olds (infants) against treating all under five years (u5-MDA), with a comparison to a placebo arm [16]. They found that treating only infants reduced infant mortality by 6% (−8% - 19%), whereas u5-MDA reduced infant mortality by a further 17% (4% - 28%) over the infant strategy. A more recent trial comparing biannual and quarterly MDA to placebo in 1–11 month olds in Mali also had negative results for the infant treatment strategy with 0% (-19% - 17%) and 7% (-15% - 25%) decreases in mortality, respectively, though the trial was also under-powered with lower mortality than expected. An analysis of mortality after MDA for trachoma control in Ethiopia [39], where MDA covered children over the age of 1 and adults, found mortality in those aged 1–5 was 47% (26% - 84%) lower than in the untreated. These findings suggest that covering wider age groups could have a strong indirect effect on mortality, indicating that disruptions to infectious diseases play a significant role.

In contrast to community-based MDA trials, individually randomised trials of prophylactic azithromycin in neonates [40] and infants [41] (less than 12 weeks old), failed to find significant reductions in mortality (19% (-17% - 44%) and -9% (-49% - 20%) for 12 and 6 month mortality, respectively). This raises the possibility that indirect effects from azithromycin MDA are important to its mechanism of action. The infant study coincided with a trial of u5-MDA [42] (which found an 18% (-2% - 33%) decrease over placebo in u5MR). They found that living in a treatment cluster for the MDA trial had no significant effect on mortality in infants, potentially undermining the concept of indirect protection from wider age-targeting, though both trials were underpowered due to low mortality [41]. A household randomised trial of u5-MDA in Mali and Burkina Faso found a 10% (-12% - 30%) non-significant increase in the risk of death or hospitalisation in the treatment group [18]. Randomisation at a household level could also have reduced impact, and azithromycin was given alongside seasonal-malaria chemoprevention in a setting with high pneumococcal conjugate vaccine coverage, which may have limited the baseline risk of mortality. Still this trial found reduced incidence of diagnosed upper respiratory tract and gastrointestinal infections but not for clinical or radiographically confirmed pneumonia, indicating that lack of power, i.e., low death rate/clinical incidence, might have masked the impact on mortality related to these diseases.

Regarding impact on specific pathogens, a 2022 review indicated that azithromycin MDA had significant impacts on diarrhoea and respiratory disease but no strong evidence for an effect on malaria or nutrition [43]. MDA of azithromycin has been linked to a short-lived reduction in diarrhoea risk, up to 39%, that returned to baseline six months post-treatment [44]. However, linking this to specific pathogens has been difficult. A sub-study of MORDOR in Niger found only a 3 percentage point (1–5) reduction in Campylobacter seroprevalence throughout the trial, with no significant findings for malarial-protozoa or other enteric pathogens [45]. Additional sub-studies looking at enteric pathogens [46] in Niger found a 64% decrease in the prevalence of *Shigella* 24 months into the intervention. Primates are the only natural hosts of *Shigella*, unlike the other studied pathogens, making long-term disruptions to transmission more likely [47].

Azithromycin MDA has also been linked to reduced respiratory infections [48]. *S. pneumoniae* (pneumococcus) are common human commensal bacteria and a leading cause of pneumonia and meningitis [49,50]. An individually randomised trial in Burkina Faso demonstrated a 43% (28% - 64%) reduction in asymptomatic carriage of pneumococcus 14 days after treatment in children who received azithromycin. Six months after the intervention ended, carriage in the treatment arm was similar to the placebo [51]. Similarly, a sub study of MORDOR in Malawi failed to find reductions in pneumococcal carriage six months after treatment [37]. A review found that out of six studies with sufficient follow-up, five observed transient decreases in the carriage of pneumococcus after azithromycin MDA that returned to baseline over time, typically within 6 months of the last administration [52]. Notably in the monitoring of nasopharyngeal carriage in children with respiratory symptoms in MORDOR in Niger, significant reductions in the carriage of *B. pertussis* and Hib were detected while not for *S. pneumoniae* [53], suggesting that MDA may have a stronger impact on pathogens with lower prevalence.

Although no conclusive evidence exists, azithromycin MDA likely reduces mortality due to a range of gastrointestinal and respiratory pathogens through immediate clearance and, if offered to wide enough age-groups at the community level, has short-lived effects on transmission, illustrated in Fig 1. Understanding how the mechanisms of action translate to pathogens common in humanitarian settings is necessary to anticipate how long and how large the reduction in mortality will be. In many humanitarian settings, the risk of faecal-oral contamination is elevated, particularly in camps before the setup of WASH facilities [4]. So, azithromycin MDA could have a greater impact on diarrhoeal diseases by preventing human-human transmission, as theorised for *Shigella*. In these settings, outbreaks of cholera and *Shigella* are a common threat, and recent research suggests that co-infection is common in diarrhoeal illness, so reduced pathogenic bacterial carriage may still be beneficial when viral pathogens drive illness [54]. The disease burden in a crisis may involve pathogens with a lower force of infection, which would experience longer indirect effects, but overcrowding could also increase transmission pressure, which would shorten the duration of the indirect effects. Thus, azithromycin MDA might need to expand eligibility to older children or adults to induce a similar effect.

## Potential risks of azithromycin MDA

Antibiotic exposure selects for and promotes the growth of antibiotic resistance in bacterial populations. A key challenge to azithromycin MDA is that it, by design, is inefficient as antibiotics are given to a significant proportion of disease-free people. Though there is no continuous exposure to azithromycin and the intervention often ends after a few rounds, azithromycin has a long half-life of elimination, meaning that sub-therapeutic doses may remain in the body for at least a week after treatment [55]. While the doses in MDA will be smaller and less frequent than those used for clinical disease, it has been theorised that sub-therapeutic levels of antibiotics can promote resistance [56]. Hence, the long-term impact of azithromycin MDA on resistance is unclear.

Table 1 summarises the evidence of macrolide and multi-drug resistance after azithromycin MDA in various pathogens. In the short-term, meaning within 6 months of a round of MDA, we see evidence of increased macrolide resistance in *S. aureus*, *E. coli*, and increased determinants of resistance in gut microbiota. Also of interest is the persistence of macrolide resistance. Resistance genes often incur a fitness cost [57], meaning the prevalence of resistance should decrease over time, but there is a risk that resistance can be fixed in the population. Few studies measured macrolide resistance in the long-term; a single study found some resistance in *S. aureus* and *E. coli*, but had no control group or baseline measurements [58]. Resistance in the gut decreased 12 months after the last MDA round [59,60]. Selection for macrolide-resistance genes may also select for resistance to other antibiotics [61]. Multi-drug resistance was observed in *S. aureus, E. coli*, and the gut microbiome in the short-term. Two studies looked at multi-drug resistance in the long-term, and both had negative findings for the gut and *C. trachomatis*. There are few studies for these pathogens and often had different follow-up times and endpoints, or lacked baseline or control groups, making meaningful inference difficult.

**Table 1:** Summary of studies measuring resistance post-MDA. Studies for C. trachomatis, S. aureus, E. coli, P. falciparum, and some of S. pneumoniae were identified by a systematic review of macrolide resistance following MDA for trachoma control [52]. Gut microbiota and remaining S. pneumoniae studies are from follow-up and sub-studies to the MORDOR trial. To aid the evaluation of the quality of each study, a key indicates whether the intervention (MDA) was randomized, R; whether the study included a control or baseline (pre-MDA) measurement of resistance, C and B; and a measurement of the quality of the study based on a modified Black and Downs checklist, Q. The checklist was used in relation to resistance endpoints/outcomes, which were secondary/additional analysis in most studies, so this should not be taken as a reflection of the quality of the study in general. See S2 Table for more information. A lack of evidence of an increase does not necessarily indicate evidence against an increase, many studies lack comparator groups or baseline measurements.

Macrolide Resistance Post Mass Drug Administration of Azithromycin			
Pathogen	Evidence of increase in resistance R: Randomized intervention (Yes/No) C: Comparison with control group (Yes/No) B: Baseline (pre-MDA) comparator (Yes/No) Q: Rating based on Downs and Black (Good/Fine/Poor)		
	In the short term (≤6 months)	In the long term (>6 months)	To other antibiotics
*C. trachomatis*	**No evidence:** 2 studies, small populations, resistance measured in trachoma (R:N C:N B:Y Q:P) [62,63]	**No evidence:** 1 study, small population (R:Y C:Y B:N Q:F) [64]	**No evidence** for resistance in doxycycline (single study, no comparator) (R:Y C:Y B:N Q:F) [64]
*S. aureus*	At 6 months, adjusted odds-ratio against non-treatment (2 years since last round) **5.22 (1.49–18.34)** for prevalence of and **15.88 (1.99–126.54)** for proportion resistant (R:Y C:Y B:N Q:F) [65]	**29% resistant at 4 years** after the last round (no comparator) (R:N C:N B:N Q:P) [58]	Increase in **clindamycin resistance** via macrolide-inducible clindamycin resistance over 6 months (R:Y C:Y B:N Q:F) [65]
*E. coli*	OR of resistant carriage **4.54 (1.83 - 10.90)** at 6 months, though **highest at 1 month** (10.38, 6.09 - 17.70) total increase of 45%. (R:N C:Y B:Y Q:P) [66] Over 6 months, exposure to MDA associated with an OR for macrolide resistance of **3.64 (2.38 - 5.78)**. (R:N C:Y B:N Q:F) [67] Both studies are from the same trial	**16% resistant at 4 years** after the last round (no comparator), similar or lower to baseline resistance in other studies. (R:N C:N B:N Q:P) [58]	Association with increase in **trimethoprim/sulfamethoxazole resistance ≤**6 months post MDA. No association found for amoxicillin, ampicillin, and chloramphenicol. (R:N C:Y B:N Q:F) [67]
*P. falciparum*	**No evidence:** 1 study (R:N C:Y B:Y Q:F) [68]	**No studies**	**No studies**
*Gut Microbiota*	MORDOR (Niger), **increased prevalence of macrolide resistance determinants at 6 months after MDA**, compared to placebo: 6th round: 7.4 (4.0 - 16.7) times [61] 5th round: 7.5 (3.8 - 23.1) times [61] 8th round: evidence of an increase, but not directly tested [47]. (R:Y C:Y B:Y Q:G) [47,61] **No increased risk from 2 more years of biannual MDA**, 0.80 (0.52 to 1.00), after 9 rounds versus 5 rounds [60]. MORDOR (Malawi), **significant increase** in proportion of macrolide-resistant bacteria [59]. (R:Y C:Y B:Y Q:G) [59,60]	MORDOR (Niger), **reduced resistance once MDA ceased**, 2.18 fold-increase (1.49 - 3.51) MDA 6 months ago versus 18 months ago. (R:Y C:Y B:Y Q:G) [60] MORDOR (Malawi), **non-significant increase** in proportion of macrolide-resistant bacteria at 12–24 months, versus baseline. (R:Y C:Y B:Y Q:G) [59]	MORDOR (Niger), No increases compared to placebo at the 4th round. (R:Y C:Y B:Y Q:G) [47] **Increased determinants of multi-drug resistance at 6th round** and non-significant at the 8th. (R:Y C:Y B:Y Q:G) [61] Longer term, around 18 months, **no increases for non-macrolide antibiotics**. (R:Y C:Y B:Y Q:G) [60] MORDOR (Malawi), evidence of an **increase in multi-drug resistance**. (R:Y C:Y B:Y Q:G) [59]
*S. Pneumoniae*	Increase observed in many trials, but results are mixed at 6 months, see Fig 3B and Fig 3C.	2 studies observe higher rates of macrolide resistant carriage (> 20%) beyond 1.5 years after the final MDA round. Few studies examined macrolide resistance in the long-term, see Fig 3A.	No changes observed in penicillin and cotrimoxazole resistance. (R:N C:N B:Y Q:P) [69] Observed increases in Clindamycin and Tetracycline after 4 rounds at 3 months. (R:Y C:Y B:N Q:F) [70] In MORDOR in Niger, no increases seen in Clindamycin, Penicillin, and others at 6 months post the 5th round. (R:Y C:Y B:Y Q:G) [71] 3.5 years post MORDOR in Malawi, increased resistance to tetracycline, penicillin, and multi-drug resistance was observed, though rates also increased in placebo groups. (R:Y C:Y B:Y Q:G) [72]

In comparison, a systematic review of resistance post-MDA for trachoma control found 12 studies looking at resistance in pneumococcus [52]. Fig 3 summarises the data from 10 of these studies [58,69,70,73–79], along with 5 studies [71,72,80–82] of resistance following trials of MDA for child survival. The majority of studies measured resistance at 6 months post MDA and few less than 6 months, where resistant carriage appeared to be higher. Six studies looked at resistance further than 6 months, three found negligible resistance [74,75,78], although they all received 2 or fewer rounds with annual spacing. A trial in Ethiopia [70] found that resistance declined after MDA ceased, though it remained at a high level 2 years after MDA, but there was no baseline reading. A follow-up to MORDOR in Malawi [72] 3.5 years after the last MDA round found higher levels of resistant carriage (between 15% and 30%) in both the treated and untreated (also treated after MORDOR) sites and in children too young to have been treated. Due to the treatment of the original trial's control group, this study lacked a natural control [83], and resistance levels were not dissimilar to those observed at baseline in this site, 21.7% and 27.6% [80]. Even so, during the original MORDOR in Malawi, an increase in resistance was also observed in the untreated arm, 32.8% after 4 rounds compared to 28% observed at baseline [80]. This potentially suggests that a spillover of macrolide resistance into the untreated group or other factors have increased resistance rates in both arms.

In short, there is evidence that azithromycin MDA promotes macrolide resistance in the short term, less than 6 months, in both gut and nasopharyngeal pathogens. Studies of pneumococcus observed both transient and longer-lasting increases in resistance. A potential explanation is the difference in size and duration of studies; more frequent rounds reaching larger populations could lead to a greater evolutionary advantage to resistance that would then persist for longer in the absence of treatment. A limitation of this data is the focus on determinants and resistance rates in carriage rather than the occurrence of antibiotic resistant clinical disease, reflecting the difficulty of continuous AMR surveillance in comparison to cross-sectional surveys of resistant carriage.

Of the diseases indicated for treatment with macrolides by the MSF medical guidelines [84] (see S1 Table), all are at risk of developing macrolide resistance, but this is rarely observed for chlamydial infections and has never been recorded for leptospirosis and relapsing fevers. Four indications have only one alternative non-macrolide antibiotic, two of which have potentially notable burdens in humanitarian crises: pertussis [85] and atypical pneumonia [86]. Other indications with high potential burdens in humanitarian settings are cholera [4], typhoid fever [4], and diphtheria [87]. Even with these diseases, there are risks to limiting treatment options. Humanitarian settings may have reduced access to medicines, so alternative antibiotics may be less viable. Some have positioned azithromycin as the alternative antibiotic for the treatment of multi-drug resistant typhoid fever [88]. Azithromycin is also used to treat bacterial STIs, so the spread of resistance in adults is also a potential concern. As of 2024, WHO recommends macrolides as a first line antibiotic for diphtheria [89]. Given rising concerns about penicillin resistance; should resistance to azithromycin also increase, treatment options may become limited.

The WHO recommendation for MDA also includes the condition that adverse effects be continuously monitored [12]. While the risk of adverse events (AEs) is unlikely to outweigh the benefits [90], exposure to azithromycin in the first month of life is associated with an increased risk of pyloric stenosis. While MORDOR protocol and the WHO recommendation does not include infants below 1 month, however, some evidence has suggested that the window of risk extends out to the first 6 weeks of life [91]. Additionally, concerns around the association between childhood exposure to macrolides and asthma have also been raised [16].

In short, AMR and AEs are serious risks of azithromycin MDA. However, these risks may not translate directly from the routine use to use in a crisis-setting. Azithromycin MDA in humanitarian settings would likely be a short-term intervention with a single round, rather than repeated biannual rounds as in routine use. This could limit the potential for macrolide resistance in this setting, as depicted in Fig 2. Additionally, other humanitarian interventions could limit the risk of the spread of AMR and macrolide-resistant infections by reducing transmission and disease severity in the long-term. However, including more age-groups should be weighed against increased AMR and age-dependent AE risk.

## Operational considerations

Mass drug administration (MDA) for malaria control has proven feasible in humanitarian crises. MDAs of artesunate/amodiaquine and dihydroartemisinin-piperaquine in the Democratic Republic of Congo [35], Sierra Leone [30], Liberia [31], and CAR [34] and have achieved high coverage of 90%, 87–96%, 90%, and 98.1% respectively. Although actual treatment initiation can be lower than coverage (52% versus 90%) [30] this is unlikely to apply in azithromycin MDA, where treatment is single dose and can be directly observed during distribution. These examples demonstrate the feasibility of MDA in crisis settings, including Ebola outbreaks that would limit access [30,31], when supported by community engagement. In a less acute situation, NTD control programmes (including MDA) have successfully treated refugee and IDP populations through the use of members of the community for access and information in displaced communities [92].

Azithromycin is simple to procure as it is a commonly used antibiotic [93] and is on the WHO’s list of essential medicines [94]. Azithromycin can be stored as a dehydrated powder with a three-year shelf-life and no particular precautions for its heat-stability before reconstitution [95], making it a candidate to stockpile ahead of crises as emergency preparedness. However, azithromycin MDAs for trachoma [96] and in randomised trials [15] have relied on donations of azithromycin from Pfizer. The cost of azithromycin, for a buyer, would be ~ $0.05 per kg of child/infant weight [97], per the MORDOR protocol and assuming minimal wastage, or ~$0.63 per child assuming a typical sub-Saharan age and weight distribution. Logistics and personnel made up 84.2% of delivery costs for MDA in a remote area of South Sudan [98], including training for community drug distributors [13], which could be reduced if integrated into other health activities. Humanitarian health actors will need little additional equipment, other than the azithromycin, safe potable water, and height-dosing poles for treating those older than 6 months [99], to complete a round of MDA. The ease of set-up and ability to pre-position means that azithromycin MDA could be quickly deployed at the start of a crisis.

Azithromycin MDA could also be administered via other platforms commonly used in humanitarian settings and could complement these interventions. Mass vaccinations are effective at mitigating increased transmission from vaccine-preventable diseases, and are often necessary to support routine immunisation programmes [9]. However, this protection takes time due to vaccines’ immunological nature, and may also be limited by malnutrition [100]. Azithromycin MDA could provide short-term relief within this period; and azithromycin MDA has been observed to cause a greater decrease in mortality in children with acute malnutrition than in those without [101]. It may be possible to initiate MDA earlier, when vaccination campaigns are delayed by shortages, cold-chain requirements or multi-dose schedules [102]. Azithromycin MDA and vaccination campaigns have synergy, with vaccination providing long-term protection that azithromycin cannot provide.

Nutritional aid can help reduce both vaccine and non-vaccine preventable disease burden by treating malnutrition, which can compromise the immune system. WASH interventions reduce the faecal-oral or vector-based transmission of pathogens. Both are vital to reduce crisis induced mortality and morbidity. Azithromycin MDA can provide initial short-term reductions, as malnutrition takes time to treat [103], as do improvements to facilities and behaviour change. Azithromycin MDA, however, would supplement and not replace these interventions, as it lacks any prospect for a long-term improvement to health.

However, it is unclear when azithromycin MDA should be initiated with no individual targeted disease. The Sphere handbook [104] offers suggestions for when to initiate reactive cholera or measles mass-vaccination programmes based on specific symptoms, e.g., deaths due to diarrhoeal disease or low vaccine coverage. The infant mortality and u5MR limits from the WHO recommendation for use of azithromycin will likely be met in an acute crisis. So, azithromycin MDA could be initiated preventively when a crisis or complex emergency has disrupted healthcare and surveillance to take advantage of its short-term effect and preempt any spikes in infant mortality [104,105].

Another consideration is the population's pre-crisis exposure to azithromycin. Populations already receiving, or scheduled to receive, MDA for trachoma control may represent a priority group. These populations may be missing doses due to disruptions, and displaced populations may have an increased risk of trachoma [106]. Implementing azithromycin MDA could address both the trachoma burden and a potential increase in mortality. Concerns regarding AMR might be weighed differently, as the population was due to receive azithromycin anyway. Operationally, this may also be more feasible as in-country stockpiles of azithromycin would be available.

Azithromycin MDA during outbreaks of diseases with notable burdens in humanitarian settings, like diphtheria or pertussis, also warrants consideration. Azithromycin is a recommended treatment and prophylaxis for contacts of both [84]. It may be feasible to supplement outbreak control; which can have poor case-ascertainment and adherence in crisis setting; and multi-dose diphtheria, tetanus, and pertussis vaccination campaigns with azithromycin MDA. Macrolides have been shown to be effective against diphtheria [107], and used to help control a pertussis outbreak [108]. However, these interventions used doses given over several days, versus the single dose recommended for improving child survival. Subinhibitory doses could promote resistance, so humanitarian actors should also consider the appropriate dose for their situation.

## Research priorities

Table 2 depicts how azithromycin MDA for a humanitarian setting should differ from MDA for child survival, and highlights some key questions for its implementation. Ongoing research will be essential to strengthen the evidence for both of these interventions. Though the conditional recommendation for routine MDAs is for sub-Saharan Africa, crises occur across the globe, the epidemiological conditions that produced the mortality benefits and AMR risks may differ in other settings. We propose that the best use-case for this intervention would be a displaced population where mortality is believed to be high or increasing but where the interventions needed to address this, nutritional aid, mass vaccination, or WASH improvements, are delayed or have yet to be deployed. Azithromycin MDA would then be deployed alongside these interventions (or before if delays are related to shortages) to provide an immediate reduction in mortality.

**Table 2:** Proposed features of azithromycin MDA in humanitarian settings in comparison to azithromycin MDA in more routine settings.

Characteristic	Azithromycin MDA to improve childhood survival	Potential use of azithromycin MDA to reduce childhood mortality in humanitarian crises	Key research questions for the implementation of azithromycin MDA in humanitarian crises	Potential sources of evidence
Initiation	Infant mortality is > 60 per 1000 live births or under-five mortality is > 80 per 1000 live Births (WHO recommendation)	As a matter of priority in acute crises or emergencies, or when under-5 mortality exceeds baseline levels or a set threshold	Would azithromycin be accessible?	Implementation study
Age coverage	1–11 months per WHO recommendation, 1–59 months per MORDOR and AVENIR trials	1-59 months for largest impact, potentially targeting older ages	Do the benefits of targeting wider age groups outweigh increased costs and AMR risk?	Translation of evidence from routine use, Modelling and cost-effectiveness
Target populations	Sub-Saharan (SSA) settings (WHO recommendation), rural communities (MORDOR and AVENIR)	Crisis affected populations, potentially internally displaced or refugee populations, likely to be smaller populations over a wider area	Feasibility of targeting displaced populations? How would evidence from SSA translate to other settings?	Translation of evidence from MDAs in crisis settings (i.e., antimalarial) Evidence from non-SSA azithromycin MDAs for trachoma control
Monitoring requirements	Infant and under-five mortality rates, adverse effects and antibiotic resistance are continuously monitored	Mortality rate monitoring might be performed. AE or AMR monitoring is unlikely to be a priority	How do the AMR risks from the routine use translate to this setting? Are AEs more of a concern in this setting?	Translation of evidence from routine use, Pilot study with cross-sectional surveys of resistance
Integration with other interventions	Implementation of existing child survival interventions, including seasonal malaria chemoprophylaxis (*sic.*) where recommended, is concurrently strengthened	Could be included with other early interventions (i.e., measles mass vaccination, malaria MDA, and nutritional screening). Synergises with interventions providing long term relief from disease (i.e., WASH and vaccination)	Could azithromycin MDA be viable in settings where trachoma is affecting displaced populations or in populations undergoing an outbreak where azithromycin is a recommended treatment or prophylaxis?	Translation of evidence from routine use, Modelling
Number of rounds	Biannual rounds	Single round	Would a single round provide sufficient protection?	Pilot study, Translation of evidence from routine use, Modelling
Potential impact on mortality	13.5% (6.7% - 19.8%) reduction in under 5 mortality (MORDOR), less impact if given to 1–11 months only (AVENIR), over 2 years	Potentially larger due to higher baseline mortality and greater burden of infectious diseases due to malnutrition/ poor sanitation. Only a single distribution/round of MDA	Do these benefits translate to humanitarian settings, with different underlying health conditions, different and more acute disease burdens, and with a reduced number of rounds?	Pilot study, Modelling
Mechanism of action	Unclear, likely some combination of bacterial respiratory and gut pathogens	Unclear, likely to affect respiratory and gut pathogens, also diseases associated with crises and displacement, i.e., diphtheria, cholera etc	Will potentially subclinical doses be sufficient to impact disease transmission? Will increased transmission rates reduce the level of indirect protection?	Pilot study, Modelling
AMR concerns	High, long-term resistance and multi-drug resistance observed in some pathogens	Unknown, potentially lower due to smaller populations and single round, although outbreaks of resistant pathogens could be a concern, particularly when resistance occurs alongside/in more virulent pathogens	Would the characteristics of a humanitarian setting increase the risk of AMR? Can this be alleviated with a smaller population and reduced number of rounds? Would existing resistance to macrolides limit impact?	Pilot study with cross-sectional surveys of resistance, Modelling Any existent monitoring of AMR infections.

Ideally, a cluster randomised trial could address many of the questions regarding mortality impact and AMR risk in humanitarian settings. However, beyond the difficulty of performing a trial in a humanitarian setting, smaller populations would prevent a cluster randomised trial and limit the power for any mortality-based outcome, and household randomisation could reduce any indirect-effects. A trial could be randomised at a camp or community level, but with few clusters stochastic events, such as an outbreak, would greatly impact mortality or mortality-averted. Longitudinal surveys, such as those used to evaluate anti-malarial MDAs, would be more feasible, though a relative lack of power would be an issue and their design would prevent distinguishing between MDA related effects and the impact of other interventions and temporal trends in mortality.

Evidence from the routine use of azithromycin for trachoma and trials on its use for child survival will also be important, particularly for concerns around AEs and to clarify the diffuse mechanism of action. If the mechanism involves directly reducing disease progression or preventing bacterial super-infections rather than an impact on disease transmission, this may limit the potential mortality reduction. Tools like mechanistic modelling can translate evidence for more specific questions, such as how reduced rounds might impact AMR development, or how wider age targeting or increased mixing might affect specific pathogens.

Due to the potential resistance and resource costs, a trial or pilot study could evaluate the AMR risks and demonstrate the feasibility of the intervention. Continuous monitoring of AMR is not a priority in an acute crisis. But AMR risk could be measured longitudinally through cross-sectional surveys of pneumococcal carriage or the gut-microbiome, before and after azithromycin MDA. The spread of AMR genes could be measured by sampling the untreated population, i.e., older adults. An additional concern relates to the possibility for MDA to create a niche to be filled by resistant and pathogenic bacteria and so increasing the rate of disease, particularly when bacterial resistance and virulence are correlated [109]. Genomic analysis of the relevant genetic elements, and modelling of resistance dynamics can help assess if the risk of this occurring is greater in crisis settings. As pre-MDA macrolide resistance might explain some variation in the impact of MDA, analyses should aim to understand this relationship in routine uses and compare to resistance profiles observed in displaced populations or the particular crisis affected setting.

## Conclusions

MDA of azithromycin has proven to be effective at reducing childhood mortality in settings with high childhood mortality by reducing infectious disease mortality. Given the need to scale health innovations in humanitarian settings, azithromycin MDA could be pre-positioned and integrated into established interventions including vaccination, improvements to water and sanitation, and interventions to address malnutrition, although more research is needed.

The exact mechanisms behind azithromycin MDA's impact on mortality are unclear and are likely to vary by setting. There is also a risk of increased macrolide resistance, which would limit treatment options for some diseases, including diseases that can have significant burdens in displaced populations. Though randomised trials might prove unable to estimate the impact on mortality, the AMR risks could still be explored. As MDA is used to improve child survival, new evidence could clarify its mechanism of action and impact on pathogens of interest, and thus its applicability for use as part of the humanitarian response.

This review is limited by its non-systematic approach to the literature. As no evidence exists for azithromycin MDA in this particular setting, we instead opted to utilise existing systematic reviews around the benefits and risks of azithromycin MDA for child-survival. However, translating evidence from non-humanitarian settings to a humanitarian setting is complicated, with potentially differing demographics, diseases, and accessibility.

Though recent studies [16,17] have cast doubt on the efficacy of the WHO recommendation for azithromycin MDA targeting children from 1-11 months, and valid concerns around AMR might prevent widespread uptake of the more efficacious treatment of children under 5, acute phase crises may present a use case for this intervention. Our findings parallel the views expressed elsewhere [110] that the future use of azithromycin MDAs, in general, must be informed by context-specific and evidence-based strategies that consider the mortality and resistance costs and their place amongst other common interventions to improve child health. Humanitarian actors considering azithromycin MDAs will need to carefully consider opportunities for monitoring benefits and risks, support future research, and help to inform future updates to the WHO guidelines.

## Supporting information

S1 Appendix Additional methodological details. Section 1 details a literature search on the use of azithromycin MDA in humanitarian settings. Section 2 details the search used in ClinicalTrials.gov to identify clinical trials relevant to azithromycin MDA. Section 3 details the conversion from a per live-birth mortality rate to per-person-time. Section 4 gives the calculations for estimating the number of crises exceeding a given mortality threshold. Section 5 gives the approach used to calculate confidence intervals for Fig 3.
(DOCX)

S1 Table Infectious diseases indicated for treatment with macrolides, any evidence of resistance to the causative agents, and whether they have a notable burden in humanitarian settings. The table includes diseases from the MSF medical guidelines [84] (AZITHROMYCIN oral, ERYTHROMYCIN oral, and CLARITHROMYCIN oral) and alternative treatments from relevant disease pages. The burden of each disease in humanitarian settings is based on the cited studies and other published work [4].
(XLSX)

S2 Table Features of the studies included in Table 1 in the main document. Trial indicates the wider trial if the study was a sub-study or secondary analysis of a larger trial/study. Randomized: was the intervention (MDA) randomized? Control group: did the study include a control (placebo group)? In some studies (*) the control was also exposed to a single round of MDA. Baseline measures: did the study report any pre-MDA measurements of resistance in this population? Treated ages: which age groups were treated during MDA? For some trachoma MDAs it was unclear (?), but the usual age group is all > 6 months of age. Measured ages: which ages were sampled for resistance as part of this study? Reported proportion of resistance: did the study report the proportion of resistance within carriage or disease? Reported prevalence of resistance (or carriage): did the study report the prevalence of resistance in the population? For reported proportion of resistance and reported prevalence of resistance, these are marked as yes if results were presented in a way where this could be calculated, e.g., line-listings with sufficient sampling denominators. Rating: methodological quality measured via the Black and Downs checklist. We skipped question 8, relating to adverse effects, and simplified question 27 into “Did they perform power/sample size calculation, at a 5% significance and 80% power to detect a clinically significant difference, and was this sample size achieved?”. As such, the total score is out of 28 and was graded for simplicity into categories as used elsewhere [111]. The checklist was applied to the measurement of AMR-related endpoints only, and does not represent the quality of the trial/study as a whole.
(XLSX)
