# Supplementary material for: Azithromycin mass drug administration to reduce childhood mortality in humanitarian crises
Source: PLOS Glob Public Health. 2026 Jul 10;6(7):e0006684. doi: 10.1371/journal.pgph.0006684 (PMC13353972; doi:10.1371/journal.pgph.0006684)
Supplement: S1 Appendix — Section 1 details a literature search on the use of azithromycin MDA in humanitarian settings. Section 2 details the search used in ClinicalTrials.gov to identify clinical trials relevant to azithromycin MDA. Section 3 details the conversion from a per live-birth mortality rate to per-person-time. Section 4 gives the calculations for estimating the number of crises exceeding a given mortality threshold. Section 5 gives the approach used to calculate confidence intervals for Fig 3. (DOCX) [file pgph.0006684.s001.docx]

# Azithromycin mass drug administration to reduce childhood mortality in humanitarian crises

# Supplement

## 1. PubMed Searches

On 09/09/2025 we performed a search for *(mass drug administration OR mda) AND (azithromycin OR childhood survival) AND (humanitarian OR emergency OR fragile)* which returned 41 results, zero of which discussed azithromycin MDA in humanitarian settings.

## 2. ClinicalTrials.gov Search

A search of clinicaltrials.gov for *azithromycin mass drug administration* in studies in children on the 09/09/2025 returned 26 trials, 12 of which focus on azithromycin to reduce mortality. 2 of these are currently active and 2 which are completed but have yet to record results. A search for *azithromycin* in trials that are currently active identified 2 more trials that explore the impact of azithromycin on mortality, though one was not mass drug administration, but azithromycin included in routine visits.

## 3. Converting between measures of mortality

The WHO recommendation for MDA to improve childhood survival gives mortality thresholds in terms of live births, i.e. u5MR of 80 per 1000 live births. Mortality surveillance (in crisis settings) is normally given in terms of person days. To convert between these two measures, we assume that the under 5 population is exponentially distributed (i.e. that the risk of death once born is constant). Hence, the probability of death before your 5th birthday is
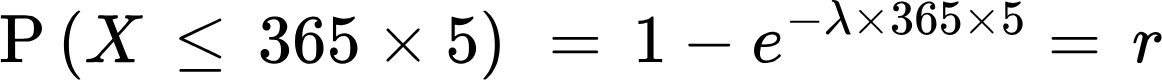
 where
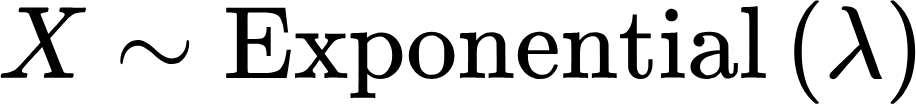
 and
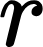
 is the rate per live birth.
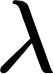
 is the rate of death per person-day, so we solve
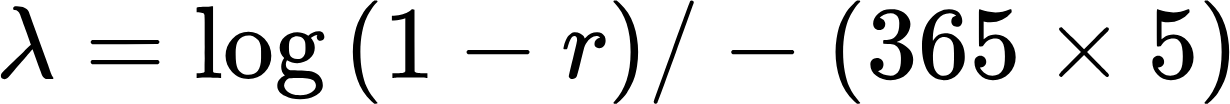
 to get the equivalent rate in person-days.

## 4. Calculating risk of exceeding mortality thresholds

A study that reviewed all mortality data from crisis from 1999-2020 reported the mean (μ) and standard deviation (σ) of the u5MR (per 10,000 person-days) for resident, IDP, and refugee populations. The database that underlies this study (Complex Emergency Database (CE-DAT)) no longer exists so we cannot use the individual data. Assuming a normal distribution of the data, we can estimate the proportion of historic crisis that exceeded a given value x, using 1 - P(X ≤ x) where X ~ Normal(μ, σ^2^), or 1 - ⁠**Φ**((x - μ)/σ).

## 5. Bayesian credible intervals for the prevalence of macrolide resistant carriage

To generate confidence bounds on the carriage of macrolide resistant pneumococcus, p_r_. We assume that:


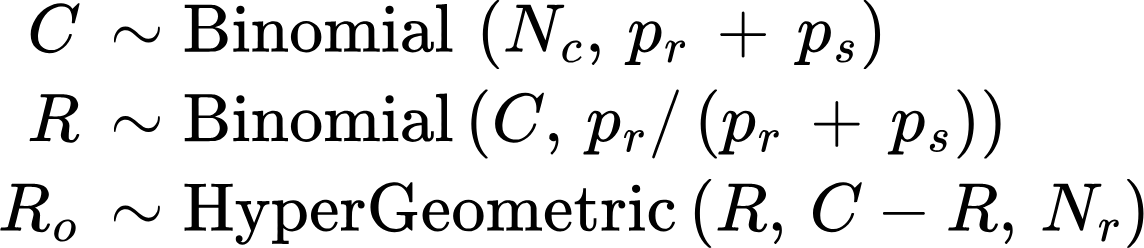


Where
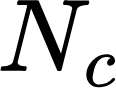
 is the number of samples taken to test for pneumococcal carriage, and
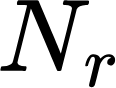
 the number of samples tested for macrolide resistance.
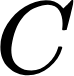
 is the number positive for pneumococcal carriage,
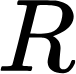
 the unobserved proportion of the sampled population that is macrolide resistant, and
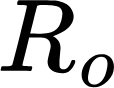
 the number of samples that are observed to be macrolide resistant. We assume flat priors on prevalence of susceptible,
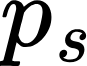
, and resistant,
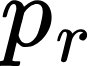
, carriage.
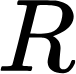
 can be numerically integrated out of the likelihood, and the credible intervals are generated using [Turing.ml](http://turing.ml/) in Julia.
